# Castleman Disease in a Patient with Common Variable Immunodeficiency

**DOI:** 10.1155/2019/5476383

**Published:** 2019-02-14

**Authors:** Luisa Ricciardi, Fabiana Furci, Antonio Ieni, Antonio Macrì

**Affiliations:** ^1^Allergy and Clinical Immunology Unit, Department of Clinical and Experimental Medicine, Medical School Hospital G. Martino, University of Messina, Messina, Italy; ^2^Department of Human Pathology in Adult and Developmental Age “Gaetano Barresi”, Unit of Pathological Anatomy, University Medical School Hospital G. Martino, University of Messina, Messina, Italy; ^3^Peritoneal Surface Malignancy and Soft Tissue Sarcoma Program, Messina University Medical School Hospital, Messina, Italy

## Abstract

Common variable immunodeficiency (CVID) is a primary immunodeficiency due to a disorder of the adaptive immune system which causes hypogammaglobulinemia and therefore an increased susceptibility to infection; noninfectious, inflammatory conditions including systemic autoimmunity and lymphoproliferative complications are also commonly associated with CVID. Castleman disease (CD) is a systemic disease clinically characterized by diffuse lymphadenopathy, splenomegaly, anemia, and systemic inflammatory symptoms. This makes CD a great mimicker of more common benign and malignant masses in the neck, chest, abdomen, and pelvis. A novel case of primary immunodeficiency (CVID) in a middle-aged woman, who developed multicentric CD (MDC) with splenomegaly, is described. The authors suggest that the onset of MCD and of the correlated splenomegaly was due to incorrect management of the hypogammaglobulinemia as immunoglobulin G (IgG) levels were not kept within normal ranges. Correct management of the hypogammaglobulinemia allowed splenectomy to be performed without any infectious surgical complications. MCD is reported for the first time in association with an adult case of CVID. The above reported case highlights the need for a timely correct diagnosis and treatment of CVID to avoid complications, which could cause recourse to splenectomy, such as in our case or development of malignancies.

## 1. Introduction

Common variable immunodeficiency (CVID) is the most common primary immunodeficiency of young adolescents and adults, which also affects children. It is characterized by low antibody levels of immunoglobulins (IgG, IgA, IgM) that cause recurrent infections, especially bacterial, which predominantly affect the respiratory and gastrointestinal tract. In the lung, it is common to find granulomatous lymphocytic interstitial lung disease (GLILD) for which it is necessary to make differential diagnosis with lymphoma. These granulomatous lymphoid aggregates may also be found at other sites. In patients with CVID, there is a greater risk of autoimmune diseases, lymphomas, and other neoplasms of the gastrointestinal tract. The treatment of this pathology is cure of the infections and chronic administration of immunoglobulins [1–3].

Castleman disease (CD), also known as angio-follicular lymph node hyperplasia, is a rare disorder that can be unicentric or multicentric. Unicentric Castleman disease (UCD) is localized and usually has an excellent prognosis. Multicentric Castleman disease (MCD) is a systemic disease clinically characterized by diffuse lymphadenopathy, splenomegaly, anemia, and systemic inflammatory symptoms [4, 5]. This makes MCD a great mimicker of more common benign and malignant masses in the neck, chest, abdomen, and pelvis as MCD masses commonly raise the suspicion of lymphoma, paraganglioma, metastatic adenopathy, solid parenchymal or neuroendocrine tumors, and infectious or inflammatory diseases [6].

MCD is associated with an increased risk of developing malignancies [7].

It may manifest itself in two forms, and patients may present with either an indolent disease and very slow progression, or an acute, fulminant disease; it has been reported to occur in HIV patients who typically have a simultaneous infection of human herpes virus 8 (HHV-8) [8].

Nevertheless, the term MCD encompasses several distinct lymphoproliferative disorders with different underlying disease pathogenesis; even histopathological features are diverse as they are seen in different clinical variants of MCD and in reactive (autoimmune/infectious) and malignant (lymphoma) context [9].

A diagnosis of MCD is made by excisional biopsy of affected lymph node tissue. Then, a computed tomography (CT) of the chest, abdomen, and pelvis should be performed to investigate the presence, or not, of adenopathy and splenomegaly. Nodal lesions in MCD more closely resemble reactive or neoplastic nodal disease and calcifications are uncommon; intralesional necrosis or fibrosis may cause a heterogenous appearance [5, 10].

We present the first clinical case of a patient with CVID who also developed MCD.

## 2. Case Report

A 51-year-old woman was diagnosed with CVID since 2000. Diagnosis was reached after her having contracted two episodes of pneumonia and developing chronic diarrhea. IVIG treatment was delivered every 45 days (4 gr/kg). Patient's IgG levels reached normal blood levels (> 700 mg/dl) with good clinical conditions. Since 2012, due to patient's personal reasons, IgG levels were not correctly kept within normal ranges; in 2017, the patient developed bilateral laterocervical lymph nodes (1 subtributary lymph node of 6.5 mm), lymph nodes in the mediastinal space (3.5 mm), and splenomegaly. Histological examination on supraclavicular and abdominal lymph node biopsies was negative for neoplasm. Clinical signs of fatigue, fevers, and night sweats as well as anemia elevated CRP levels, and hepatosplenomegaly was present. The patient was diagnosed with MCD and referred to our clinical immunology unit due to severe hypogammaglobulinemia and splenomegaly.

Blood count detected hypochromic microcytic anemia, mild neutropenia, and thrombocytopenia. The study of lymphocyte subpopulations showed an inverted CD4/CD8 T-cell ratio due to the numerically expansion of CD8^+^ T-cells. Immunoglobulin levels were low: IgG 345, IgA 2, and IgM 4 mg/dl. Wright agglutination test, markers of hepatitis B, hepatitis C, HIV, HHV8, tumor markers, serum and urine immunofixation, and fecal antigen* H. Pylori* were normal.

IVIG treatment was started at 5 g/Kg maintaining IgG levels > 700 mg/dl as well as i.v. iron therapy.

**Figure 1:**
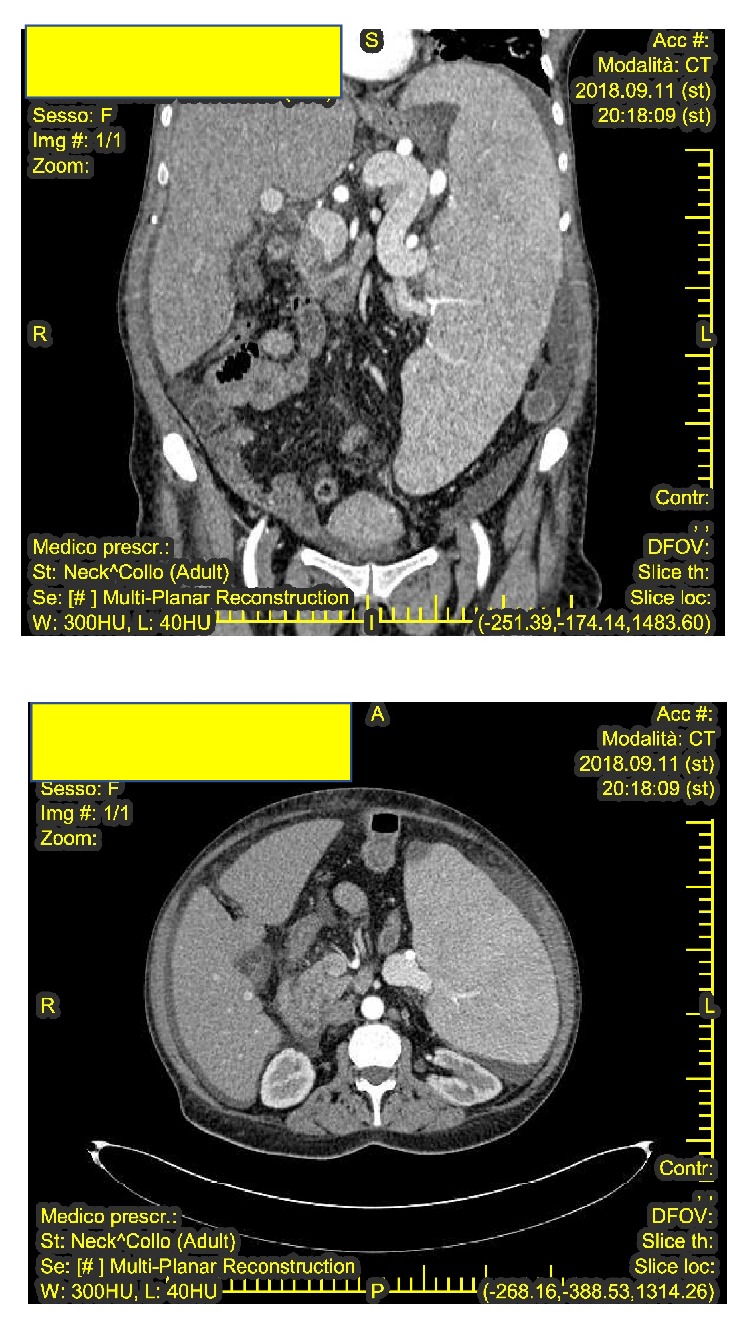
Thoracic -abdominal CT with contrast medium which confirmed the presence of adenopathy in the mediastinal space.

A complete abdomen ultrasound detected hepatomegaly (large wing 22 cm), splenomegaly (greater than 30 cm), with a lesion at the splenic pole of 26 mm, increased portal vein (20 mm), thick gastric and mesenteric walls, and modest free spillage in the right and left iliac fossa. A thoracic-abdominal CT with contrast medium showed the presence in both lungs of numerous occurrences of parenchymal thickening with nodular appearance, some confluent, with irregular morphology, and contours. The examination also highlighted the presence of bronchiectasis. Numerous paraaortic and iliac-obturator lymph nodes with a short axis of about 12 mm were identified. Other lymph nodes were identified in the celiac site and along the small gastric curvature. The liver, increased in volume, did not show focal lesions. Port vein ectasia (24 mm) and splenic vein ectasia (25 mm) were highlighted (Figure 1).

**Figure 2:**
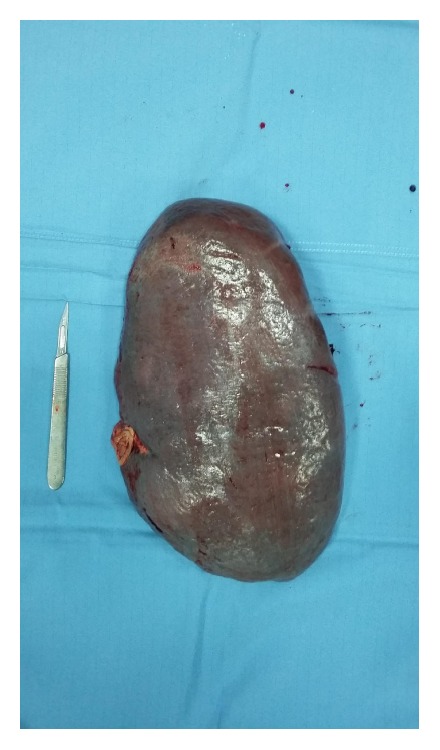
Patient's spleen after splenectomy.

Surgical counselling recommended splenectomy. As it was not an emergency surgery, in order to prevent any infectious surgical complication, IgG levels were maintained over 700 mg/dl for 2 months before splenectomy (Figure 2).

**Figure 3:**
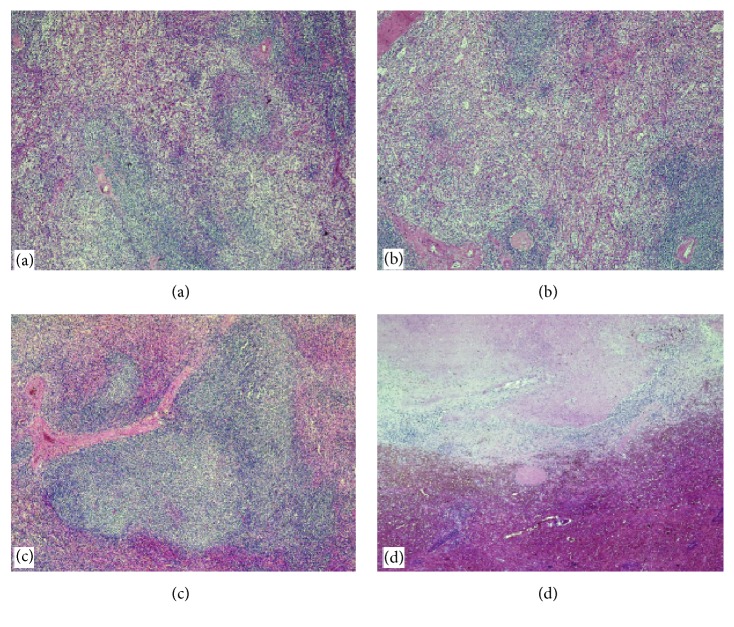
Histopathological analysis showed a diffuse plurifocal nodular white pulp hyperplasia characterized by an admixture of lymphocytes and aggregates of macrophages (H&E, 100x), follicular lymphoid hyperplasia (H&E, 100x) and pseudonodular necrotic area surrounded by prominent haemorrhagic parenchyma (H&E, 100x).

Spleen biopsies were performed, which showed a predominant lymphocytic infiltration (Figure 3).

A further thoracic-abdominal CT scan was performed three months after surgery, which showed a reduced size of the numerous paraaortic and iliac-obturator lymph nodes with a short axis of about 8 mm.

## 3. Discussion

CVID is a pathology which includes different phenotype presentations. Among the various phenotypes, the clinical case presented represents one of considerable importance. This phenotype of CVID and Castleman's disease is characterized by recurrent or chronic infections, lymphoid nodular hyperplasia, hepatosplenomegaly, progressive chronic lung disease with bronchiectasis, and increased risk of lymphoma [2, 11]. Furthermore, the case we describe is the first case of adult CVID associated with Castleman's disease as in literature only a pediatric case was previously reported [12]. The case evolution is suggestive for a role of hypogammaglobulinemia in the development of MCD as a CT scan after six months of correct IVIG treatment showed a decrease in size of lymph nodes. Therefore, we hypothesize that the multicentric adenopathy with splenomegaly was a consequence of inappropriate treatment of CVID. The constant low levels of blood immunoglobulins most likely, as already reported, hyperstimulated the immune system causing a lymphoproliferative disorder with adenopathy and splenomegaly [13].

Idiopathic MCD, usually diagnosed after excision biopsy and comprehensive work-up of symptomatic lymph node masses, has been reported to be characterized by an exaggerated systemic inflammatory response secondary to a cytokine storm involving Interleukin-6 (IL-6).

A therapeutic approach for MCD is, in fact, anti-IL-6 therapy siltuximab [14].

Surgeons may also have an important role in the diagnostic work-up of MCD [15]; in the case we describe, MCD with severe splenomegaly secondary to CVID was the reason why the patient was referred to the surgeon for splenectomy. Correct management of the hypogammaglobulinemia allowed splenectomy to be performed without any infectious surgical complications.

In the light of the different phenotype presentations of CVID, further research on this pathology is increasingly necessary [16]. In the case we reported, we hypothesize that MCD associated with CVID was secondary to an incorrect maintenance of IgG circulating blood levels. CVID treatment, focused on maintaining IgG levels within normal ranges, is essential to avoid consequences due to infections and lymphoproliferative disorders.
